# Chemotherapy Shows a Better Efficacy Than Endocrine Therapy in Metastatic Breast Cancer Patients with a Heterogeneous Estrogen Receptor Expression Assessed by ^18^F-FES PET

**DOI:** 10.3390/cancers14143531

**Published:** 2022-07-20

**Authors:** Yizhao Xie, Xinyue Du, Yannan Zhao, Chengcheng Gong, Shihui Hu, Shuhui You, Shaoli Song, Xichun Hu, Zhongyi Yang, Biyun Wang

**Affiliations:** 1 Department of Breast Cancer and Urological Medical Oncology, Fudan University Shanghai Cancer Center, Shanghai 200032, China; vermouth1993@126.com (Y.X.); 14211230018@fudan.edu.cn (Y.Z.); gcckino@163.com (C.G.); 20211230025@fudan.edu.cn (S.H.); 21211230028@m.fudan.edu.cn (S.Y.); huxichun2017@163.com (X.H.); 2 Department of Oncology, Shanghai Medical College, Fudan University, Shanghai 200032, China; xinyuedu2020@163.com (X.D.); 16211230029@fudan.edu.cn (S.S.); 3 Department of Nuclear Medicine, Fudan University Shanghai Cancer Center, Shanghai 200032, China; 4 Center for Biomedical Imaging, Fudan University, Shanghai 200032, China; 5 Shanghai Engineering Research Center of Molecular Imaging Probes, Shanghai 200032, China

**Keywords:** breast cancer, ER heterogeneity, ^18^F-FES PET/CT, diagnosis, treatment pattern

## Abstract

**Simple Summary:**

About 10–20% of breast cancer patients have a heterogeneous estrogen receptor expression. The diagnosis and treatment strategy remains controversial in these patients, especially regarding the metastatic pattern. The aim of our study was to investigate the occurrence and properties of estrogen receptor heterogeneity and to evaluate the following treatment efficacy among a certain group of metastatic breast cancer patients. We found the novel ^18^F-FES PET/CT method could identify patients with estrogen receptor heterogeneity, and chemotherapy showed a better efficacy compared with endocrine therapy in these patients. Our findings could give valuable suggestions to physicians and researchers in clinical practice.

**Abstract:**

Background: The heterogeneity of estrogen receptor (ER) expression has long been a challenge for the diagnosis and treatment strategy of metastatic breast cancer (MBC). A novel convenient method of ER detection using ^18^F-fluoroestradiol positron emission tomography/computed tomography (^18^F-FES PET/CT) offers a chance to screen and analyze MBC patients with ER uncertainty. Methods: MBC patients who received ^18^F-FES PET/CT were screened and patients with both FES positive (FES+) and negative (FES-) lesions were enrolled in this study. Progression-free survival (PFS) was estimated using the Kaplan–Meier method and was compared using the log-rank test. Results: A total of 635 patients were screened and 75 of 635 (11.8%) patients showed ER uncertainty; 51 patients received further treatment and were enrolled in this study. Among them, 20 (39.2%) patients received chemotherapy (CT), 21 (41.2%) patients received endocrine-based therapy (ET), and 10 (19.6%) patients received combined therapy (CT + ET). CT showed a better progression-free survival (PFS) compared with ET (mPFS 7.1 vs. 4.6 months, HR 0.44, 95% CI 0.20–0.93, *p* = 0.03). CT + ET did not improve PFS compared with either CT or ET alone (mPFS 4.4 months, *p* > 0.2). All three treatment options were well tolerated. Conclusions: ^18^F-FES PET/CT could identify patients with ER heterogeneity. Patients with bone metastasis are more likely to have ER heterogeneity. Patients with ER heterogeneity showed better sensitivity to CT rather than ET. Combined therapy of CT + ET did not improve the treatment outcome.

## 1. Introduction

Breast cancer is the most common malignancy accounting for 30% of female cancers and is the second leading cause of cancer death in women [1]. Estrogen receptor positive (ER+) breast cancer constitutes more than 70% of all breast cancers [2]. Normally, ER+ breast cancer patients have lower rates of recurrent disease and a better prognosis compared with other molecular subtypes [3]. However, more and more research points out that the heterogeneity of ER could affect the treatment response and overall prognosis [4,5,6]. Furthermore, whether traditional endocrine therapy (ET) is still applicable in tumors with ER uncertainty remains controversial.

Novel methods of detection for ER heterogeneity are warranted because multiple pathological punctures are often infeasible, especially for metastatic patients. ^18^F-fluoroestradiol (^18^F-FES) positron emission tomography/computed tomography (PET/CT) is a non-invasive, molecular imaging technique to observe and quantify ER status in vivo [7]. ^18^F-FES is now widely used in the diagnosis and treatment prediction of breast cancer patients [8,9]. Moreover, studies have demonstrated that ^18^F-FES uptake correlates well with ICH scoring for ER [10,11]. It has been reported that a conspicuous number of patients present with discordant ER expression between primary tumor and metastasis, and ^18^F-FES PET/CT is used to reveal the existence and prognostic effects of ER heterogeneity [7,12,13,14].

Previous studies indicate that patients with low positive ER (ER expression 1–10%) have unique molecular features and thus are more sensitive to chemotherapy (CT) rather than endocrine therapy (ET) [15]. We considered whether a similar situation happens in patients with ER heterogeneity.

Few studies focus on ER heterogeneity among MBC patients because of the difficulties in ER detection among multiple lesions. Therefore, the purpose of our study is to investigate the occurrence and properties of ER heterogeneity using ^18^F-FES PET/CT and to evaluate the following treatment efficacy among a certain group of MBC patients.

## 2. Methods

### 2.1. Patients

We screened all MBC patients who received ^18^F-FES PET/CT in Fudan University Shanghai Cancer Center from 2017–2021. Patients who had both FES positive (FES+) and negative (FES−) lesions were enrolled in this study. Patients who did not receive further treatments or who had incomplete medical records were excluded.

MBC was defined as unresectable, recurrent, or metastatic breast cancer. Medical and PET/CT data were collected retrospectively from the electronic medical database system.

Fudan University Shanghai Cancer Center Ethics Committee and Institutional Review Boards approved this clinical study. All of the techniques and methods were conducted in accordance with the Declaration of Helsinki and the relevant guidelines. This research is registered under clinicaltrials.gov (NCT05392985).

### 2.2. ^18^F-FES PET/CT Imaging

All of the chemicals were obtained from commercial sources and were used without further purification. The MMSE precursor and the authentic ^18^F-FES was purchased from Jiangsu Huayi Chemical Co, Ltd. (Suzhou, Jiangsu, China). ^18^F-FES was prepared according to the published methods [16]. To prevent ^18^F-FES false-negative results, ER antagonists were discontinued for a minimum of 5 weeks before the study. The use of aromatase inhibitors was allowed [17]. All of the patients received an injection of approximately 222 MBq (6 mCi) of ^18^F-FES over 2 min. Scanning consisted of a whole-body PET/CT examination (2–3 min per table position) initiated 1 h after the administration of the tracer on a Siemens biograph ^16^HR PET/CT scanner. The transaxial intrinsic spatial resolution was 4.1 mm (full width at half maximum) in the center of the field of view. The PET/CT data acquisition protocol was as follows: CT scanning was first acquired from the proximal thighs to the head using a low-dose technique (120 kV, 80–250 mA, pitch 3.6, rotation time 0.5 ms). Immediately after the CT scan, a PET emission scan that covered the identical transverse field-of-view was obtained. We used a Gaussian-filter iterative reconstruction method to reconstruct the PET images. The coregistered images were displayed on a workstation.

### 2.3. Image Interpretation

A multimodality computer platform (Syngo, Siemens, Knoxville, TN, USA) was utilized for image review and manipulation. Two experienced board-certified nuclear medicine physicians evaluated the images independently and reached a consensus in cases of discrepancy. Lesions in ^18^F-FES PET/CTs were identified using paired ^18^F-FDG PET/CT images. Semiquantitative analysis of the tumor metabolic activity was obtained using SUV normalized to body weight. The maximum SUV (SUVmax) for each metastatic lesion was recorded by manually placing an individual ROI around each tumor on all consecutive slices that contained the lesion on coregistered and fused transaxial PET/CT images. We used a cut-off value of SUVmax ≥ 1.82 or SUVmean ≥ 1.21 to dichotomize the results as either ER positive and negative [18,19]. Patients with both ER positive and negative lesions were defined as having ER heterogeneity.

### 2.4. Outcome Measurements

The primary outcome measurement was PFS of different treatment groups (ET, CT, and ET + CT); secondary measurements were the incidence and characteristics of ER heterogeneity as well as treatment safety. PFS was defined as the time from the first dose of treatment to disease progression or death from any cause. The National Cancer Institute Common Terminology Criteria for Adverse Events (CTCAE) version 4.03 was used to evaluate safety. Response Evaluation Criteria in Solid Tumors (RECIST) version 1.1 was used for the tumor response: complete response (CR), partial response (PR), stable disease (SD), and progressive disease (PD).

### 2.5. Statistical Analysis

The quantitative data were presented as medians (range) or number of patients, and the categorical data were shown as counts (percentage). Descriptive statistics were used in the clinicopathologic characteristics and the Chi square test was used to compare between groups. Descriptive statistics were also used to depict the secondary outcomes. The survival analyses were evaluated with Kaplan–Meier method, and the hazard ratios (HRs) and corresponding 95% confidence intervals (CIs) were estimated using the Cox proportional hazard model. Prognostic factors were investigated using the Cox regression model with a 95% confident interval in both the univariate and multivariate models. A *p* value less than 0.05 was considered statistically significant. All statistical analyses were managed using SPSS (IBM) version 23.0 or R language (R i386 4.0.2).

## 3. Results

### 3.1. FES Results and Treatment Options

**Figure 1:**
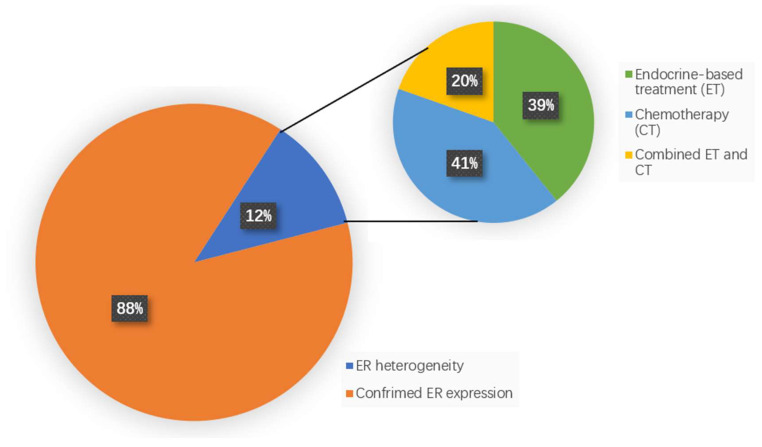
Incidence and treatment pattern of patients with ER heterogeneity.

A total of 635 MBC patients who received ^18^F-FES PET/CT were screened, and 560 patients had confirmed ER positive or negative expression, while 75 of 635 (11.8%) patients showed ER heterogeneity. Among the 75 patients with a heterogeneous ER expression, 51 patients received further treatment, met our inclusion criteria, and were enrolled in our study. With regards to the treatment alternatives, 20 (39.2%) patients received chemotherapy (CT), 21 (41.2%) patients received endocrine-based therapy (ET), and 10 (19.6%) patients received combined therapy (CT + ET) (Figure 1).

### 3.2. Patient Characteristics

The baseline patient characteristics between the three treatment groups are summarized in Table 1. The median ages of the ET and CT groups were 55, and the ET + CT group was 48. A majority of patients had surgery before, while 14%, 10%, and 20% of patients were de novo stage IV patients in the ET, CT, and ET + CT groups, respectively. Most of the patients were in good status. Visceral metastasis occupied about half of patients, while the majority of patients had bone metastasis (over 80%). The median treatment lines were the first and second lines for three groups. Overall, no significant differences were observed in the baseline characteristics between the three groups.

### 3.3. Treatment Efficacy

The most frequently applied (>20%) CT regimens were the combined treatment of two chemotherapy regimens (55%) and capecitabine (30%); ET regimens were aromatase inhibitor/fulvestrant (42.8%) and CDK4/6 inhibitors plus aromatase inhibitor/fulvestrant (28.6%); and the ET + CT regimens were capecitabine plus aromatase inhibitor/fulvestrant (90%).

**Figure 2:**
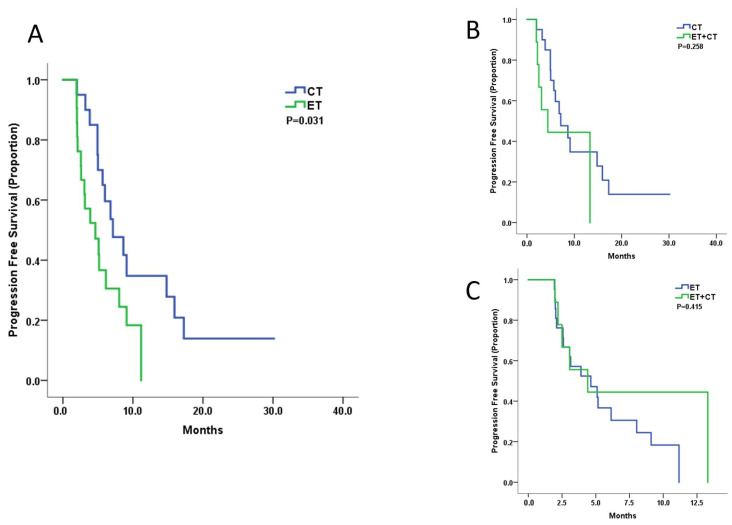
Kaplan–Meier curves for progression-free survival by treatment arm: (**A**). ET versus CT; (**B**) CT versus ET + CT; (**C**) ET versus ET + CT.

**Figure 3:**
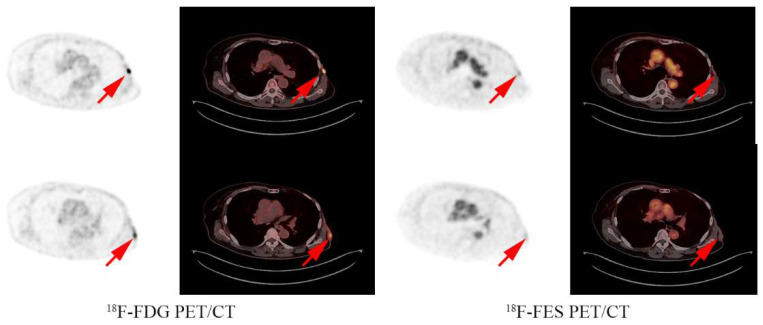
Analysis examples: A 73 year old female had an FES positive lesion in the chest wall and a FES negative lesion in the axillary lymph nodes and received fulvestrant treatment with a PFS of 4 months.

**Figure 4:**
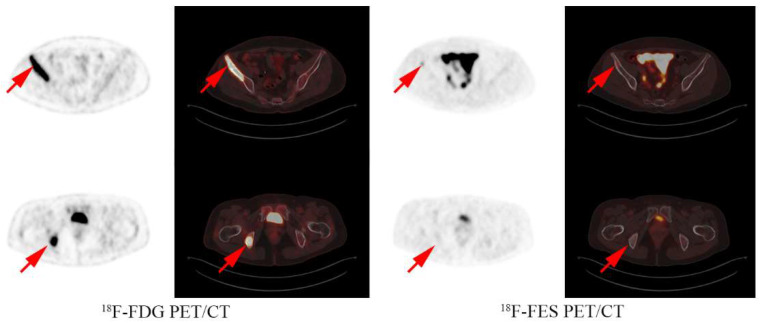
Analysis examples: A 53 year old female had both FES positive and negative lesions in the bone and received capecitabine treatment with a PFS of 15 months.

At the median 18-month follow-up, 38 of the 51 patients experienced progressive disease. The median PFS of the CT group was 7.1 months (95% CI 3.8–10.5), the ET group was 4.6 months (95% CI 1.8–7.4), and the ET + CT group was 4.4 months (95% CI 0.5–8.3). CT showed better mPFS compared with ET (HR 0.44, 95% CI 0.20–0.93, *p* = 0.03, Figure 2A). CT + ET did not improve the PFS compared with either CT (HR 1.32, 95% CI 0.81–2.17, *p* = 0.26) or ET alone (HR 0.66, 95% CI 0.24–1.81, *p* = 0.42) (Figure 2B,C). The multivariate analysis showed CT treatment as an independent prognostic factor, even after balancing the DFI, age, visceral metastasis, number of metastatic sites, and prior MBC treatment lines (adjusted HR 0.46, 95% CI 0.22–0.98, *p* = 0.043). The analysis examples of the CT and ET group are shown in Figure 3 and Figure 4.

### 3.4. Safety

We collected and analyzed the grade 3/4 adverse events of different treatment groups (Table 2). Only one diarrhea and leukopenia case were seen in the ET group, while more hematologic toxicity and peripheral neurotoxicity were observed in the CT group. Palmar-plantar erythrodysesthesia syndrome was seen in both the CT and ET + CT groups. Overall, the three groups all showed an acceptable safety profile without a significant statistical difference (*p* = 0.14).

## 4. Discussion

This study uncovered the real-world prevalence of MBC patients with ER heterogeneity using the ^18^F-FES PET/CT method and evaluated the strategy, efficacy, and safety of the following treatments. As far as we know, this is the first investigation in ER heterogeneity using ^18^F-FES PET/CT method.

In terms of the incidence of ER heterogeneity, among all 635 MBC patients who received an FES scan, 11.8% patients had ER heterogeneity, which was lower than a previous study showing a 32.4% change in ER status in recurrent tumors compared with the primary tumors [20]. It is reasonable that for traditional study, only one metastatic site could be evaluated using the IHC method, thus increasing the false positive and false negative results. Interestingly, the incidence is similar to patients with a ER low expression reported before (6–11%), which might suggest a similar biological feature between ER heterogeneity and a low ER expression [15].

We found a majority of patients with ER heterogeneity had bone metastasis (86%). Previous preclinical study demonstrated that the osteogenic niche could reversibly reduce ER expression and activities in bone micrometastases, thus leading to endocrine resistance [21]. Our data, to some extent, proved this phenomenon in a clinical setting, which should further remind physicians to double check the ER status of MBC patients with bone metastasis in the case of ER heterogeneity in clinical practice.

With regards to treatment alternatives, about 40% patients received ET or CT, and the remaining 20% patients chose ET combined with CT. The current data fully indicated the clinical dilemma in patients with ER heterogeneity. Endocrine therapy remains to be the first choice for the first line treatment of luminal type MBC patients, both in guidelines and clinical practice [22,23]. However, among patients with a low ER expression, endocrine therapy seems to be less effective [15]. Traditionally, we tend not to use combined treatment of ET and CT because of the antagonism of chemotherapy-induced cytotoxicity by antiestrogens [24]. However, more and more research hint to the synergistic effect of ET plus CT, making it a relatively reasonable choice [25].

The current study revealed the superiority of CT over ET with regards to PFS. As for ER positive MBC patients, real-world data from Holland indicate that initial ET had an overwhelming advantage compared with CT in both PFS and OS settings [26]. Another propensity score analysis revealed that ET was not inferior to CT in first line treatment of ER positive MBC patients [27]. Our results showed the opposite results, which further demonstrated that patients with ER heterogeneity had a completely different biological behavior and treatment response from ER positive patients. On the other hand, besides the fact that ET is not suitable for ER/PR negative patients, researchers also found that patients with a ER low expression had worse treatment outcomes for ET and overall survival compared with a ER high expression [15]. This phenomenon, to some extent, suggests that patients with ER heterogeneity might be similar to those with a low ER expression or negative ER expression.

In our study, the combined treatment of ET and CT did not improve the treatment efficacy compared with either ET or CT alone. Although ET plus CT is not a common treatment option clinically, some studies have explored its feasibility. First, the SWOG-8814 trial revealed that the sequential use of cyclophosphamide, doxorubicin, fluorouracil (CAF), and tamoxifen (CAF-T) was better than concurrent CAF and tamoxifen (CAFT), although not reaching statistical significance [28]. However, the exploratory analysis of the TEXT/SOFT study showed that the concurrent use of triptorelin with chemotherapy was not associated with a significant difference in breast cancer-free interval compared with sequential triptorelin post-chemotherapy [29]. In an advanced setting, a phase II trial used fulvestrant with metronomic capecitabine on luminal-type MBC patients and found a mPFS of 15 months, which gave confidence on this treatment pattern [25]. Our study suggested little benefit gained from ET in patients with ER heterogeneity.

As expected, more 3/4 adverse events were observed in the CT group, although this did not reach statistical difference, partly because of the sample limits. Capecitabine plus aromatase inhibitors/fulvestrant were well tolerated in the ET + CT group. All adverse events were reversed after symptomatic treatment. Notably, patients needed to suspend or reduce the treatment dose after the diagnosis of grade 3 peripheral neurotoxicity, which were mostly caused by capecitabine according to previous study [30].

In conclusion, this study revealed the incidence and treatment pattern of patients with ER heterogeneity using the ^18^F-FES PET/CT method. Patients with bone metastasis are more likely to have ER heterogeneity. Patients with ER heterogeneity showed better sensitivity to CT rather than ET. The combined therapy of CT + ET did not improve the treatment outcome. Capecitabine-based treatments were well tolerated.

As this study is exploratory, more randomized controlled trials (RCT) are warranted to give more evidence regarding treatment among ER heterogenous patients. In this era of precision medicine, more and more novel methods in multidisciplinary cooperation will bring about the best benefit to patients.

## 5. Conclusions

This study revealed the incidence and treatment pattern of patients with ER heterogeneity using the ^18^F-FES PET/CT method. Patients with bone metastasis are more likely to have ER heterogeneity. Patients with ER heterogeneity showed better sensitivity to CT rather than ET. The combined therapy of CT + ET did not improve the treatment outcome. Capecitabine-based treatments were well tolerated. Our findings not only provided a novel way of detecting ER heterogeneity, but also suggested a better efficacy of CT compared with ET among patients with ER heterogeneity, which could help physicians to make decisions.

## Figures and Tables

**Table 1:** Baseline characteristics of the patients.

Characteristics	Endocrine-Based Therapy (ET) N = 21 n (%)	Chemotherapy (CT) N = 20 n (%)	ET + CT N = 10 n (%)	*p* Values
Median age	55	55	48	0.39
(range)	(29–73)	(39–70)	(32–68)	
Age > 48	16	15	7	0.93
Median disease-free interval	3	3	2	0.76
(range)	(0–13)	(0–12)	(0–15)	
De novo stage IV	3 (14)	2 (10)	2 (20)	
ECOG score				
0–1	19 (90)	19 (95)	9 (90)	0.84
≥2	2 (10)	1 (5)	1 (10)	
Number of metastatic sites				
1	10 (48)	11 (55)	6 (60)	0.78
2	8 (38)	7 (35)	4 (40)	
≥3	3 (14)	2 (10)	0 (0)	
Metastatic sites				
Visceral	11 (52)	10 (50)	4 (40)	0.81
Liver	3 (14)	3 (15)	1 (10)	0.92
Lung	10 (48)	6 (30)	2 (20)	0.26
Bone	18 (85)	16 (80)	10 (100)	0.32
Median treatment lines	1	1	2	0.12
(range)	(1–4)	(1–6)	(1–5)	

**Table 2:** Adverse events (grade 3/4).

Adverse Events (Grade 3/4)	ET N = 21 n (%)	CT N = 20 n (%)	ET + CT N = 10 N (%)
Diarrhea	1 (4.8)	0	1 (10)
Leukopenia	1 (4.8)	4 (20)	0
Anemia	0	1 (5)	0
Thrombocytopenia	0	1 (5)	
Palmar-plantar erythrodysesthesia syndrome	0	1 (5)	1 (10)
Peripheral neurotoxicity	0	2 (10)	0
All	2 (9.5)	7 (35)	2 (20)

## Data Availability

The datasets generated and/or analyzed during the current study are not publicly available due to hospital policy, but are available from the corresponding author upon reasonable request.
